# Effectiveness and safety of non-vitamin K antagonist oral anticoagulant in the treatment of patients with morbid obesity or high body weight with venous thromboembolism: A meta-analysis

**DOI:** 10.1097/MD.0000000000035015

**Published:** 2023-09-08

**Authors:** Hongxia Zhang, Hua Xie, Xiaoyan Wang, Zonghong Zhu, Feng Duan

**Affiliations:** a Department of Neurology, Sixth Medical Center, General Hospital of The People’s Liberation Army, Haidian District, Beijing, China; b Healthcare Department 1, Second Medical Center, General Hospital of The People’s Liberation Army, Haidian District, Beijing, China; c Department of Emergency, Sixth Medical Center, General Hospital of The People’s Liberation Army, Haidian District, Beijing, China.

**Keywords:** high body weight, meta-analysis, morbid obesity, non-vitamin k antagonist oral anticoagulant, venous thromboembolism, vitamin K antagonists

## Abstract

**Background:**

Venous thromboembolism (VTE) poses a significant health risk to patients with morbid obesity or high body weight. Non-vitamin K antagonist oral anticoagulants (NOACs) are emerging treatments, but their effectiveness and safety compared with vitamin K antagonists (VKAs) in this population are yet to be thoroughly studied.

**Methods:**

We conducted a systematic review and meta-analysis, adhering to the Preferred Reporting Items for Systematic Reviews and Meta-Analyses guidelines. Four electronic databases were searched for relevant studies comparing the efficacy and safety of NOACs and VKAs in treating patients with VTE with a body mass index > 40 kg/m^2^ or body weight > 120 kg. Eligible studies were scored for quality using the Newcastle-Ottawa Scale.

**Results:**

Thirteen studies were included. The meta-analysis results showed that compared to VKAs, NOACs significantly decreased the risk of VTE occurrence (odds ratio = 0.72, 95% CI: 0.57–0.91, *I*^2^ = 0%, *P* < .001) and were associated with a lower risk of bleeding (odds ratio = 0.74, 95% CI: 0.58–0.95, *I*^2^ = 0%, *P* < .05). Subgroup analysis showed that in the cancer patient subgroup, both risks of VTE occurrence and bleeding were lower in the NOAC group than in the VKA group. In patients without cancer, the risk of VTE was significantly lower in the NOAC group.

**Conclusion:**

NOACs appear to be more effective and safer than VKAs in patients with morbid obesity or a high body weight with VTE. However, further large-scale randomized controlled trials are required to confirm these findings.

## 1. Introduction

Venous thromboembolism (VTE), including deep vein thrombosis (DVT) and pulmonary embolism, is a major global health concern.^[1]^ VTE has a high incidence and mortality rate, with an annual incidence of 1 to 2 cases per 1000 individuals globally.^[1–3]^ pulmonary embolism has been identified as the third leading cause of cardiovascular disease-related death, superseded only by stroke and coronary heart disease.^[4]^

The escalating global obesity epidemic requires further attention. Besides being in a prethrombotic state, obesity is an independent risk factor for VTE, amplifying the risk by a factor of 6.2.^[5,6]^ Altered body physiology in obese individuals affects the pharmacokinetics and pharmacodynamics of therapeutic drugs, including increased distribution volume and clearance rate.^[7]^ This complex interplay between obesity and VTE necessitates the development of tailored therapeutic strategies for patients with obesity. Non-vitamin K antagonist oral anticoagulants (NOACs) have been touted as a new hope in the realm of VTE treatment, achieving the status of first-line agents.^[8]^ However, due to increased body mass, the plasma concentration of NOACs may be diluted, resulting in a weaker anticoagulant effect. This dilution effect is attributed to the increased protein binding rate and distribution volume in the obese population.^[9]^

In light of these concerns, the international society on thrombosis and hemostasis (ISTH) issued a recommendation in 2016 against the use of NOACs in patients with a body mass index (BMI) of > 40 kg/m^2^ (representing morbid obesity) or a body weight of > 120 kg.^[10]^ However, these guidelines have not been universally followed in clinical settings because of the perceived benefits of NOACs, including improved patient compliance.^[11]^ Notwithstanding, the foremost therapeutic approach for VTE in patients with morbid obesity or high body weight (>120 kg) is anticoagulation.^[12]^ In recent updates to the ISTH guidelines, the use of rivaroxaban or apixaban has been endorsed in these patients.^[11,13]^ However, the guidelines have limited supporting data for apixaban and explicitly advise against using dabigatran and edoxaban.

While previous meta-analyses have attempted to shed light on this topic, the small number of included studies and varied results have led to persistent uncertainties regarding the efficacy and safety of NOACs in this population. Moreover, since the latest meta-analyses were published, new studies have emerged, underscoring the need for an updated synthesis of available evidence. Thus, in this study, we performed a comprehensive systematic review and meta-analysis to evaluate the effectiveness and safety of NOACs in patients with morbid obesity or high body weight with VTE, providing a valuable reference for clinical decision-making.

## 2. Materials and Methods

Throughout the systematic review and subsequent dissemination of our findings, we ensured compliance with the Preferred Reporting Items for Systematic Reviews and Meta-Analyses guidelines.^[14]^ As the data utilized in this study were obtained from published sources, obtaining informed consent or ethical approval was deemed unnecessary. A systematic search of relevant studies was conducted by 2 researchers who independently assessed their eligibility, extracted the data, and evaluated the quality of the research. The 2 researchers were obligated to arrive at mutual agreement and address any areas of discordance.

### 2.1. Search strategy

On May 6, 2023, a search was conducted using 4 electronic databases, namely PubMed, Embase, Web of Science, and Cochrane Library, without any temporal restrictions. Lexicons and grammatical structures were tailored to conform to the specifications of the database. The specific search terms in PubMed were: (“NOAC” [Title/Abstract] odds ratio (OR) “novel oral anticoagulants” [Title/Abstract]) AND (“venous thromboembolism” [Title/Abstract] OR “VTE” [Title/Abstract]) AND (“obesity” [Title/Abstract] OR “high body weight” [Title/Abstract]) AND (“treatment” [Title/Abstract] OR “effectiveness” [Title/Abstract] OR “safety” [Title/Abstract]). No restrictions were imposed on the language used in this study. The reference lists of pertinent articles were manually screened to identify any potential additional records.

### 2.2. Inclusion criteria

Studies included in the systematic review were required to meet the following criteria: participants had a BMI > 40 kg/m^2^ or body weight > 120 kg and were diagnosed with VTE; Intervention: Participants in the experimental group must be undergoing treatment with NOACs. Participants in the control group must be undergoing treatment with vitamin K antagonists (VKAs); Study types: Only randomized controlled trials (RCTs) and cohort studies will be included.

The exclusion criteria were as follows; Literature that has been published multiple times; Documents that contain incomplete or unclear analytical data and inconsistent outcome indicators; Documents that exhibit poor quality and lack original data; and Types of literature that fall under the category of nonsystematic reviews, including case reports, commentaries, expert opinions, and narrative reviews.

### 2.3. Data extraction

Literature screening and data extraction will be conducted independently by 2 evaluators and subsequently verified through cross-checking. In case of any inconsistencies encountered during this procedure, the reviewers involved ought to engage in a discussion to resolve the matter and, if needed, seek the input of a third reviewer. Data extraction for this meta-analysis encompasses several key areas. First, general information, such as the author(s) of the study, year of publication, type of study, clinical characteristics, sample size, and follow-up duration, will be collected. Second, the primary efficacy and safety endpoints were the incidence of VTE and bleeding, respectively. The definition of bleeding will adhere to the guidelines set by the International Society on Thrombosis and Haemostasis.^[15]^ In instances where the published report lacks pertinent data, communication is established with the primary researchers of the original study via electronic mail to solicit unreleased data.

### 2.4. Quality assessment

The assessment of the studies incorporated in our meta-analysis will be subjected to rigorous evaluation by 2 independent reviewers using the Newcastle-Ottawa Scale (NOS).^[16]^ The NOS is a widely recognized instrument that assesses research studies according to 9 distinct elements distributed among 3 fundamental domains: selection, comparability, and outcome. The utilization of these categories allows for the evaluation of potential sources of bias that may be inherent in the studies conducted. After a thorough assessment, a numerical rating between 0 and 9 was assigned to each study to indicate the level of quality. The scoring system for the evaluation of studies was as follows: studies that received a score ranging from 0 to 3 were classified as low-quality research, whereas those that scored between 4 and 6 were considered to be of moderate quality. Finally, studies that achieved a score ranging from 7 to 9 were categorized as high-quality studies.

### 2.5. Statistical analyses

The variability across studies was evaluated using chi-square statistics and characterized by the magnitude of *I*^2^. A value of 0% for *I*^2^ suggests the absence of heterogeneity, whereas values exceeding 50% indicate a significant degree of heterogeneity. The present study employed symmetry of the funnel plot and Egger test to investigate the presence of publication bias in meta-analyses comprising a minimum of 10 eligible papers. In the event of an asymmetrical funnel plot, researchers conducted a hypothetical imputation of negative unpublished studies to assess the potential impact of publication bias on the estimated effects. In all statistical tests, a significance level of *P* < .05 was deemed to be statistically significant. Data analysis was performed using Stata version 17 (StataCorp, College Station, TX).

## 3. Results

### 3.1. Search results and study selection

**Figure 1.**
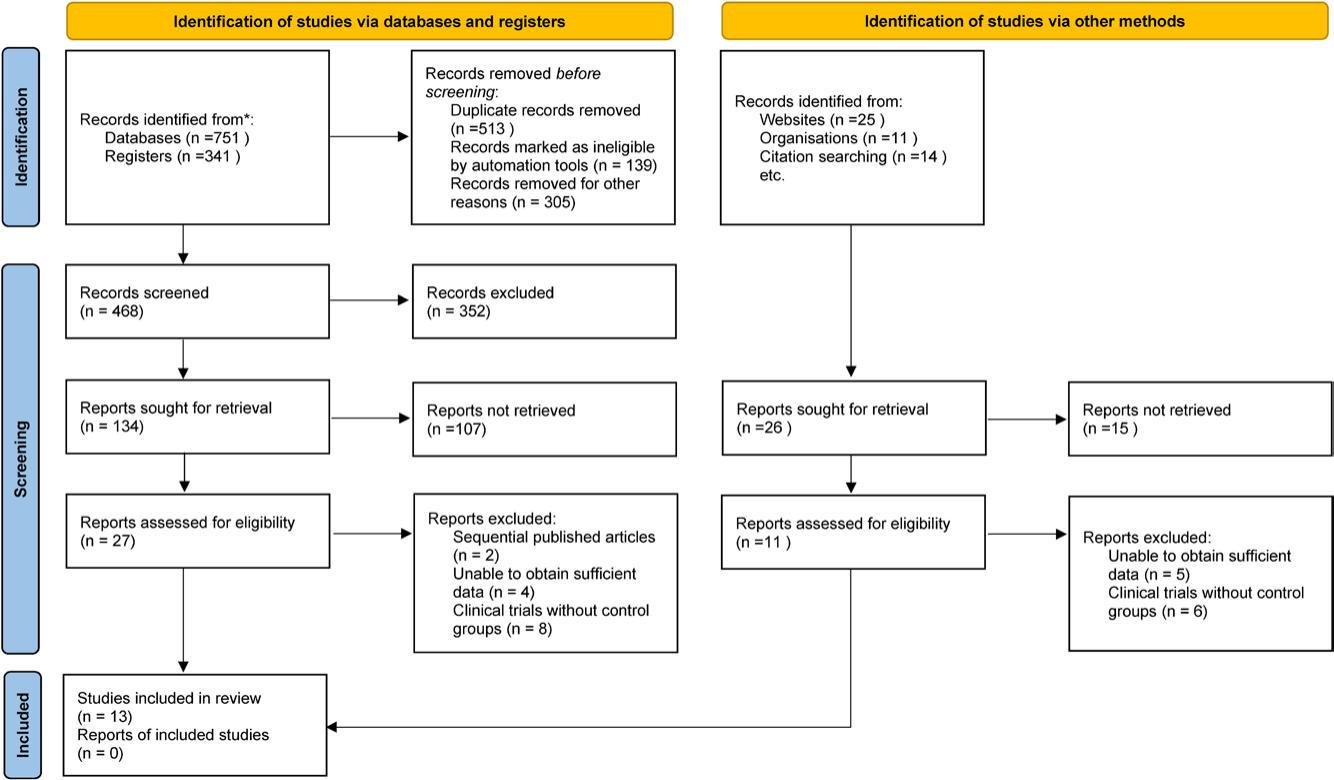
Selection process of included studies.

A total of 1425 relevant literature sources were identified by conducting an initial search of the electronic databases. Following the elimination of redundant literature, a thorough examination of titles and abstracts, and rigorous adherence to the established inclusion and exclusion criteria, 27 relevant pieces of literature were procured. Fourteen of these were deemed ineligible for further analysis. Ultimately, 13 studies were ultimately included.^[17–29]^ Figure 1 depicts the process and outcomes of literature screening.

### 3.2. Study characteristics

The studies included in this meta-analysis were primarily cohort studies (11 of 13), with only 2 RCTs. These studies were published between 2016 and 2022, with a focus on recent research. The studies generally included patients with BMI >40 kg/m² or weight > 120 kg. The NOACs groups in these studies were composed of various anticoagulants including Apixaban, Rivaroxaban, Dabigatran, and Edoxaban, whereas the VKAs group primarily consisted of warfarin, with 1 study also including heparin, and 2 others including Low Molecular Weight Heparin. The percentage of cancer patients varied across the studies, from none reported in some studies to up to 37.6% in the study conducted by Wysokinski in 2020. The follow-up durations also varied, ranging from 6 to 12 months, and were not reported in some studies (Table 1).

**Table 1:** Characteristics of studies included in the meta-analysis.

Included studies (first author)	Year	Study type	Inclusion criteria	NOAC group	VKA group	Percentage of cancer patients (%)	Follow-up duration (mo)
Crouch	2022	Cohort Study	BMI > 40 kg/m² or weight > 120 kg	Apixaban (n = 314)	Warfarin (n = 785)	NOAC: 8.6, VKA: 5.4	12
Scott	2022	Cohort Study	BMI > 40 kg/m²	Rivaroxaban, apixaban, dabigatran (n = 129)	Warfarin (n = 118)	6	-
Weaver	2022	Cohort study	BMI > 40 kg/m² or weight > 120 kg	Rivaroxaban (n = 487)	Warfarin (n = 785)	NOAC:7.3, VKA:5.3	12
Cohen	2021	Randomized controlled trial	BMI > 40 kg/m²	Apixaban (n = 126)	Heparin, Warfarin (n = 137)	NOAC: 3.2, VKA: 2.2	6
Costa	2021	Cohort study	BMI > 40 kg/m²	Rivaroxaban (n = 1697)	Warfarin (n = 1697)	0	12
Perino	2021	Cohort study	Weight > 120 kg	Rivaroxaban, apixaban, edoxaban, dabigatran (n = 2663)	Warfarin (n = 4271)	6	-
Patil	2020	Cohort study	BMI > 40 kg/m² or weight > 120 kg	Rivaroxaban, apixaban, dabigatran (n = 79)	Warfarin (n = 73)	NOAC: 3.8, VKA:5.5	NOAC: 13.9, VKA: 24.1
Perales	2020	Cohort study	BMI > 40 kg/m² or weight > 120 kg	Rivaroxaban (n = 47)	Warfarin (n = 62)	NOAC: 3.6, VKA:7.6	12
Quan	2020	Cohort study	Weight > 120 kg	Rivaroxaban, apixaban, dabigatran (n = 109)	Warfarin, low molecular weight heparin (n = 78)	NOAC: 2.8, VKA: 20.5	12
Wysokinski	2020	Cohort study	Weight > 120 kg	Rivaroxaban, edoxaban (n = 108)	Warfarin, low molecular weight heparin (n = 81)	37.6	6
Kushnir	2019	Cohort study	BMI > 40 kg/m²	Rivaroxaban, apixaban (n = 199)	Warfarin (n = 167)	6.5	-
Almeida Sa	2019	Cohort study	Weight > 120 kg	Rivaroxaban, apixaban, edoxaban (n = 71)	Warfarin (n = 62)	0	12
Di Nisio	2016	Randomized controlled Trial	Weight > 120 kg	Rivaroxaban (n = 159)	Warfarin (n = 144)	-	6.7

“-” indicates not mentioned.

BMI = body mass index, NOAC = Non-vitamin K antagonist oral anticoagulant, NOS = Newcastle-Ottawa Scale, VKA = vitamin K antagonist.

### 3.3. Results of quality assessment

The methodological quality of each included study was evaluated using the Newcastle-Ottawa Scale. The distribution of NOS scores was as follows: 2 studies achieved a score of 7, 5 studies reached 8 points, and the remaining 6 studies garnered the highest score of 9. Notably, none of the studies implemented blinding or exhibited any evidence of allocation concealment. Furthermore, all studies were free from observable funding biases. No issues of incomplete outcome data, premature termination bias, or imbalances in baseline characteristics were identified in any of the studies. The specific risk of bias and corresponding ratios for each study are presented in Table 2.

**Table 2:** The quality assessment according to NOS of each cohort study.

Study	Selection				Comparability	Outcome			Total score
	Representativ-eness of the exposed cohort	Selection of the non -exposed cohort	Ascertainment of exposure	Demonstration that outcome	Comparability of cohorts	Assessment of outcome	Was follow-up long enough	Adequacy of follow-up of cohorts	
Crouch	★	★	★	★	★★	★	★	★	9
Scott		★	★	★	★★	★	★	★	8
Weaver	★	★	★	★	★★	★	★	★	9
Cohen	★	★	★	★	★★	★		★	8
Costa	★	★	★	★	★★	★	★	★	9
Perino	★	★	★	★	★	★	★	★	8
Patil	★	★		★	★	★	★	★	7
Perales	★	★	★	★	★★	★	★	★	9
Quan	★	★	★	★	★★	★	★	★	9
Wysokinski	★		★	★	★	★	★	★	7
Kushnir		★	★	★	★★	★	★	★	8
Almeida Sa	★	★	★	★	★★	★	★	★	9
Di Nisio	★	★	★	★	★★	★		★	8

NOS = Newcastle-Ottawa scale.

### 3.4. The results of meta-analysis

**Figure 2.**
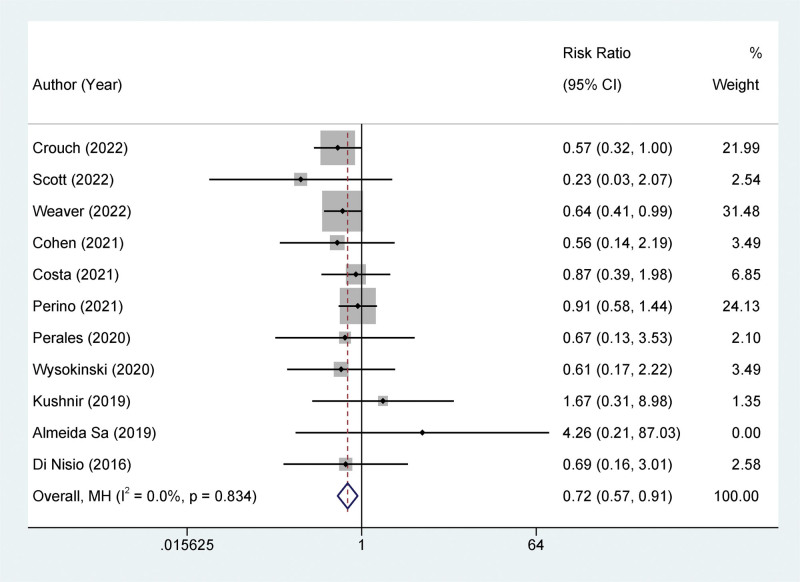
Forest plot of the incidence of Venous Thromboembolism (VTE) in patients using different types of anticoagulants.

The results from our meta-analysis indicated low heterogeneity among all included studies (*I*^2^ ≤ 50%), suggesting the use of a fixed-effects model for our analysis. To assess VTE risk, we integrated data from 11 studies,^[17–24,26,28,29]^ encompassing a total of 14,302 patients. Compared with VKA, NOAC significantly decreased the risk of VTE occurrence (OR = 0.72, 95% CI: 0.57–0.91, *I*^2^ = 0%, *P* < .001, Fig. 2).

**Figure 3.**
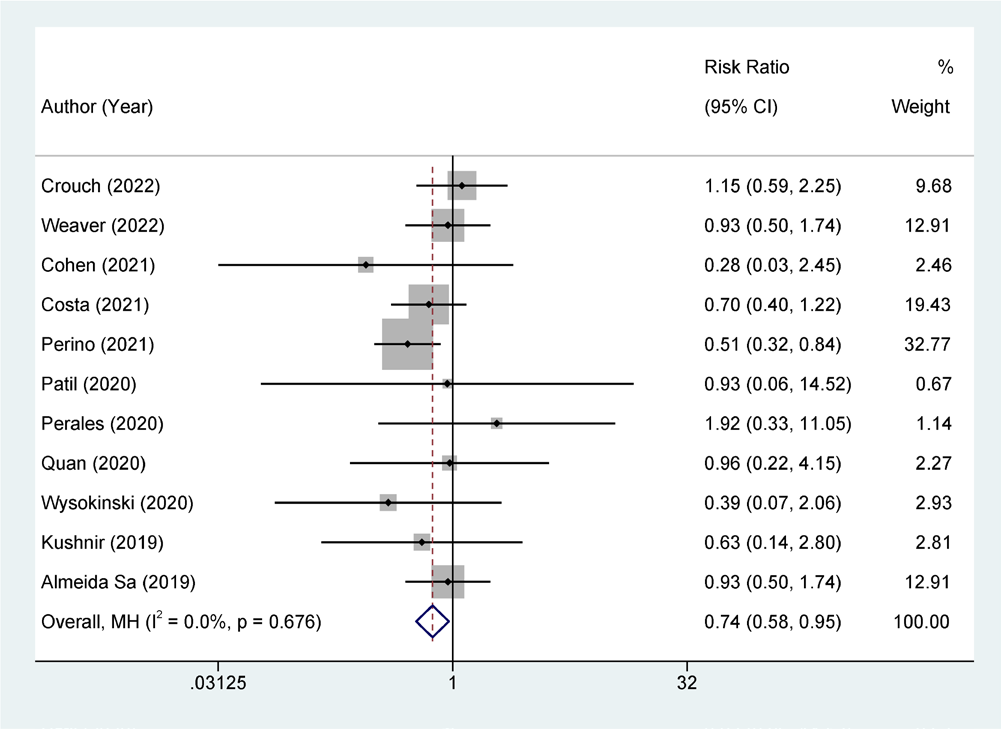
Forest plot of the incidence of major bleeding in patients using different types of anticoagulants.

Furthermore, we incorporated data from 11 studies^[17,19–28]^ into our analysis of bleeding risk, with 14,098 patients included. Relative to VKA, NOAC use was associated with a lower risk of bleeding (OR = 0.74, 95% CI: 0.58–0.95, *I*^2^ = 0%, *P* < .05; Fig. 3).

### 3.5. The results of subgroup analysis

Upon subdividing the data into subgroups according to the presence of cancer, we found that within the cancer patient subgroup, the risks of VTE occurrence (OR = 0.60, 95% CI: 0.43–0.84, *P* < .001) and bleeding (OR = 0.68, 95% CI: 0.55–0.77, *P* < .001) were both lower in the NOAC group than in the VKA group. In the non-cancer patient subgroup, the NOAC group demonstrated a lower risk of VTE occurrence (OR = 0.68, 95% CI: 0.54–0.87, *P* < .001) than the VKA group, while the risk of bleeding (OR = 0.71, 95% CI: 0.52–1.01, *P* = .055) was not significantly different. Given that none of the included studies reported the use of direct thrombin inhibitors, we only performed a subgroup analysis on the efficacy and safety of Factor Xa inhibitors. Compared to the VKA subgroup, the Factor Xa inhibitor subgroup had a lower risk of VTE occurrence (OR = 0.61, 95% CI: 0.48–0.76, *P* < .001), but the risk of bleeding (OR = 0.86, 95% CI: 0.62–1.21, *P* = .265) was not statistically significant (Table 3).

**Table 3:** The results of subgroup analysis.

Subgroup	No. of studies	NOAC group events/total	VKA group events/total	*I*² (%)	Effect model	OR (95% CI)	*P* value
Cancer patients							
VTE	5	49/1078	147/1847	0	Fixed effect	0.60 (0.43–0.84)	.001
Bleeding	7	38/1270	66/2001	0	Fixed effect	0.68 (0.55–0.77)	.001
Non-cancer patients							
VTE	6	105/4918	179/6459	29	Fixed effect	0.68 (0.54–0.87)	<.001
Bleeding	4	53/4630	101/6197	0	Fixed effect	0.71 (0.52–1.01)	.055
Factor Xa inhibitors							
VTE	9	124/3204	271/3917	0	Fixed effect	0.61 (0.48–0.76)	<.001
Bleeding	8	58/3049	97/3776	0	Fixed effect	0.86 (0.62–1.21)	.265

NOAC = non-vitamin K antagonist oral anticoagulant, OR = odds ratio, VKA = vitamin K antagonistm, VTE = venous thromboembolism.

### 3.6. Publication bias

**Figure 4.**
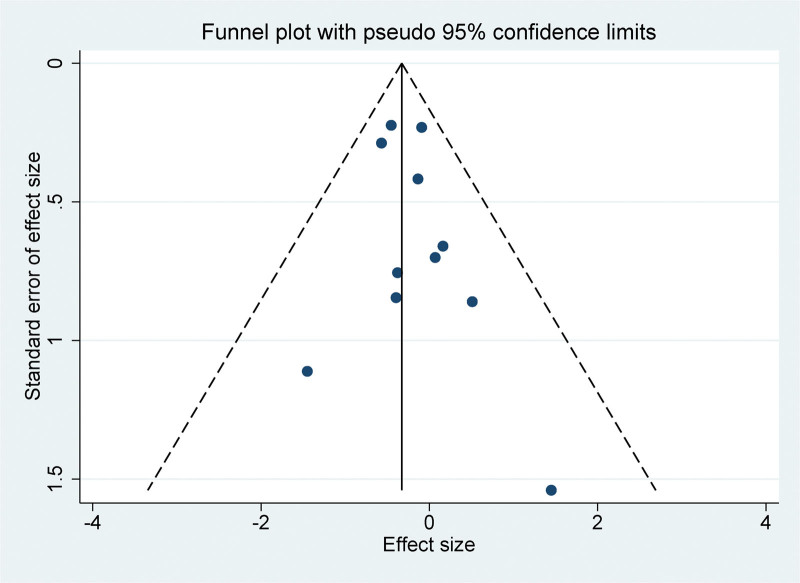
Funnel plot for publication bias in all included studies.

The funnel plots generated from the observed study exhibited symmetry and no statistically significant evidence of publication bias was identified in the corresponding funnel plots (Fig. 4). Egger linear regression test was used to evaluate the presence of publication bias in the meta-analyses across various variables. The results indicated that no significant publication bias was observed (*P* > .05), which further supports the validity and reliability of the meta-analysis outcomes.

## 4. Discussion

Obesity, a global pandemic of chronic disease, is estimated to affect over 50% of the global population by 2030, with the prevalence of morbid obesity surpassing 10%.^[30]^ Anticoagulation remains the central strategy for preventing VTE in morbidly obese patients.^[31,32]^ NOACs offer distinct advantages over warfarin, including fewer drug-drug interactions and less influence from dietary intake.^[33,34]^ They also present predictable bioavailability and pharmacokinetics and are usually administered at fixed doses without the need for regular coagulation monitoring.^[8,35]^ Nevertheless, obese patients demonstrate significant differences in pharmacokinetic characteristics compared to those with a normal weight.^[7,36,37]^

Studies on pharmacokinetics have shown that compared to patients with normal weight, the peak concentrations of rivaroxaban in patients with high body weight are either similar or reduced, while the peak concentrations of apixaban and dabigatran are decreased.^[38–41]^ This introduces considerable uncertainty regarding the application of fixed-dose NOACs in morbidly obese patients. A previous meta-analysis by Katel et al included 5 studies and indicated that the risk of VTE and bleeding with NOACs was similar to that with Vitamin K antagonists.^[42]^ However, this meta-analysis was limited by the fact that 1 study included patients with morbid obesity identified by the International Classification of Disease coding rather than calculating BMI from height and weight data, comprising approximately 88% of the patients in the meta-analysis.^[43]^ Another meta-analysis by Elshafei et al^[44]^ showed similar bias and unreliable results owing to the inclusion of the same studies.

The current study addresses the effectiveness and safety of NOACs in this population through a comprehensive meta-analysis. Compared to published meta-analyses, our study included a larger number of studies (13 in total), 6 of which were published in the past 1 to 2 years. By excluding lower-quality studies, we minimized the bias and ensured low heterogeneity in the study population. Our findings demonstrated a lower risk of VTE and bleeding in patients with morbid obesity or high body weight receiving NOACs than in those receiving VKAs. This suggests the superior effectiveness and safety of NOACs over VKAs in these patients. Moreover, our meta-analysis excluded studies involving cancer patients who are known to have an elevated risk of VTE and bleeding. Even after excluding these studies, our subgroup analysis confirmed that NOACs reduced the risk of VTE and bleeding compared with VKAs in patients with morbid obesity or high body weight, further cementing NOACs as the optimal choice for anticoagulation therapy in these patients.

VTE is a common complication in cancer patients.^[45]^ Recently, the International Initiative on Thrombosis and Cancer and the American Society of Clinical Oncology guidelines have recommended rivaroxaban, edoxaban, and low molecular-weight heparin as first-line options for cancer-related VTE.^[31,32]^ Studies suggest that only approximately 12% of cancer patients treated with warfarin can achieve therapeutic international normalized ratios.^[33]^ A meta-analysis by Wang et al^[34]^ showed the superior effectiveness and safety of NOACs compared to VKAs in patients with cancer-related VTE. However, given the underrepresentation of morbidly obese patients in these studies, our subgroup analysis specifically included studies of patients with cancer and morbid obesity and showed a reduced risk of VTE and bleeding with NOACs compared to VKAs, although the difference in bleeding risk was not statistically significant. Published phase III RCTs indicate that apixaban, dabigatran, rivaroxaban, and edoxaban present similar or lower risks of VTE and bleeding than VKAs in patients with normal weight.^[35–38]^ Our meta-analysis, featuring subgroup analysis by different types of NOACs, supports these findings, demonstrating the superiority of Factor Xa inhibitors over VKAs in terms of efficacy and safety in patients with morbid obesity or high body weight. Given that the majority of NOACs included in our subgroup analysis were rivaroxaban or apixaban, it remains uncertain whether the findings can be generalized to edoxaban. There was no separate analysis of the efficacy and safety of direct thrombin inhibitors in the studies included in our meta-analysis; therefore, no subgroup analysis was performed for these agents. Future studies should focus on the clinical use of edoxaban and dabigatran in patients with morbid obesity to guide clinicians in making more precise anticoagulant choices.

Our meta-analysis has some limitations. First, most studies included were retrospective cohort studies. Second, most studies did not provide information on the doses of NOACs used. Third, the primary results of this meta-analysis are applicable only to patients with morbid obesity or high body weight and may not be appropriate for other patient populations. Fourth, all studies compared NOACs with VKAs, with no study comparing different types of NOACs.

## 5. Conclusion

NOACs are more effective and safer than VKAs in patients with morbid obesity or a high body weight. However, further large-scale RCTs are required to confirm the effectiveness and safety of NOACs in this population.

## Acknowledgements

We appreciate the technical support provided by the hospital.

## Author contributions

**Conceptualization:** Hongxia Zhang.

**Data curation:** Hua Xie.

**Formal analysis:** Hongxia Zhang.

**Investigation:** Xiaoyan Wang.

**Methodology:** Zonghong Zhu.

**Project administration:** Hua Xie.

**Writing – review & editing:** Feng Duan.
